# Reinkes'edema: immunoexpression study of fibronectin, laminin and colagen IV in 60 cases by imunohistochemical techniques

**DOI:** 10.1016/S1808-8694(15)30543-7

**Published:** 2015-10-19

**Authors:** Regina Helena Garcia Martins, Maria Aparecida Domingues, Alexandre Todorovic Fabro, Norimar Hernandes Dias, Marcela Ferreira Santana

**Affiliations:** 1 Associate Professor - Medical School of Botucatu - Unesp. Head of the Speech and voice ward, Professor of Otorhinolaryngology - Paulista State University -Unesp, Botucatu Campus; 2 PhD. Professor of Pathology - Unesp; 3 Resident Physician - Pathology, Unesp; 4 PhD. Professor of Otorhinolaryngology, Unesp-Botucatu. MD. ENT; 5 Medical Student Medical School of Botucatu, Unesp

**Keywords:** type iv collagen, laryngeal edema, fibronectins, immunohistochemistry, laminin

## Abstract

Reinke's edema is chronic laryngeal disease in which the superficial layer of the lamina propria is expanded by thick mucus, giving it a gelatin aspect. The disease is directly related to smoking and more frequent in women, who end up having a lower tone of voice. Its histological characteristics cannot always distinguish it from other benign lesions of the larynx for which additional histological techniques are necessary.

**Aim:**

to study the immunoexpression of fibronectin, collagen IV and laminin in Reinke's edema by immunohistochemical technique. Prospective study.

**Materials and methods:**

histological blocks of 60 cases of surgical Reinke's edema were saved, submitted to new cross-sections and to immunohistochemical reactions for fibronectin, laminin and collagen IV by the Avidin-Biotin-Peroxidase method. Fragments of five normal vocal folds were used as control, removed during autopsy. All patients were chronic smokers and adults– 50 women and 10 men.

**Results:**

the immunoexpression of fibronectin, collagen IV and laminin was more important in the endothelium of blood vessels (68.33%, 76.66%, 73.33%, respectively) and less relevant in the basement membrane (25.0%, 5.0% and 3.3%, respectively).

**Conclusions:**

the immunoexpression of fibronectin, laminin and of collagen IV in the basal membrane of Reinke's edema was not relevant, with a predominance of these antibodies in the endothelium of blood vessels.

## INTRODUCTION

Reinke's edema is a chronic laryngeal disease in which the superficial layer of the lamina propria, also called Reinke's space, occupied by a thick mucous, providing the vocal folds with a gelatinous and myxomatous aspect. It happens to chronic smokers, predominantly to women - the reason is unknown so far. For some authors, such fact is due to the great concern of women with the progressive transformation that happens to their voices, which becomes lower, often times being confused with male voices^1^, ^2^, ^3^.

Reinke's edema has been included among the benign exudative lesions of the larynx, together with vocal polyps and nodules, because of histopathology similarities between these lesions, in which there is a predominance of submucosal edema, justifying the homogeneity found in pathology reports^4^,^5^. Nonetheless, these three lesions have different pathophysiological mechanisms and endoscopic aspects.

Many etiological factors are involved in the development of benign laryngeal lesions, making it possible to identify the predominance of some of them, such as smoking, in the development of Reinke's Edema and speech trauma in vocal nodules. Regarding vocal polyps, the identification of a more predominant etiological factor is more difficult

Histological studies have exhaustively tried to differentiate each one of the benign laryngeal lesions; nonetheless, the alterations found are common to the three lesions, without specificity, and there are varied degrees of epithelial hyperplasia, basal membrane thickening, corium edema, inflammatory infiltrate and vessel prolipheration^6^, ^7^, ^8^. Remacle et al.^5^ studied the histological characteristics (using hematoxylin & eosin) of the basal membrane and lamina propria epithelium of 163 vocal fold benign lesions, and noticed that in the nodules there was clear basal membrane thickening, edema and corium fibrosis and frequent epithelium parakeratosis; in polyps, corium edema, fibrosis, dilatation and vascular neo-proliferation predominated and none or very mild basal membrane thickening; in Reinke's edema, we noticed basal membrane thickening, corium edema, vessel congestion and fibrosis. Dikkers, Nikkels^7^ studied the histological alterations of the three laryngeal lesions using different dyes (H&E, Verhoeff-van Gienson, Masson Trichrome and Alcian blue after treating the tissue with hyaluronidase, summarizing the main findings as follows: in vocal polyps there is a predominance of fibrin deposits on the lamina propria, signs of hemorrhage, increase in the number of vessels, iron deposit and vascular thrombosis; in Reinke's edema, basal membrane thickening, edematous lakes, extravascular erythrocytes, vessel wall thickening predominate; in the nodules, the most prevalent alterations were basal membrane thickening, no hemorrhage and no edematous lakes.

Volic et al.^8^, reviewed 10 histology slides of Reinke's edema dyed by hematoxylin/eosin and Azan-Mallory, and noticed a normal or hyperplastic epithelium, lamina propria disarray, basal membrane thickening, vascular dilatation and edema.

The lack of histological specification among the many laryngeal lesions has led to many ultrastructural studies, aiming at finding structural details in order to differentiate these disorders. Therefore, a marked and constant finding in vocal nodules is the deposit of subepithelial heterogeneous material, rarely seen in other lesions. Other marked ultrastructural findings, however without establishing specificity among the lesions, are the focal areas of basal membrane detachment and interruptions and the widening of cell junctions, causing structural alterations in the desmosomes. All these findings are interpreted by the authors as a response to speech trauma by the epithelium, being clearer in the lesions associated to high vocal demand, as it happens with vocal nodules^9^, ^10^, ^11^.

In an immunohistochemical assay, Gray^6^ and Courey^10^ reported an important increase in fibronectin and collagen IV in vocal nodules. As far as polyps and Reinke's edema are concerned, there was a lack of fibronectin in the lamina propria, no basal membrane alterations and reduction in type IV collagen. The large amount of fibronectin was associated with the speech trauma, since it is a glycoprotein which participates in the tissue repair process.

As we saw, the results from the morphological analysis showed that vocal nodules have peculiar traits which differentiate them from the other lesions, thus being necessary to carry out additional studies involving the other laryngeal lesions so as to better understand its morphological characteristics.

## MATERIALS AND METHODS

This research project was approved by the Ethics in Research with Human Beings Committee of the institution where it was carried out under protocol number 190/06. After its approval, we carried out a retrospective review of the medical charts of all the patients with clinical and endoscopic diagnosis of Reinke's Edema, previously seen at the Voice Disorders Ward of this institution, from 2001 through 2007, and we excluded those charts with incomplete data. Thus, we selected 72 cases of Reinke's Edema, of which 60 had been submitted to surgery. Data review on the charts of these 60 patients revealed that 50 of them were females and 10 were males, nine of them were between 20 and 40 years of age, 44 were between 41 and 60 years and seven had more than 61 years of age. All the patients were chronic smokers and only 12 drank alcoholic beverages frequently. Vocal abuse was noticed in 20 cases, nasosinusal symptoms in 16 and gastroesophageal symptoms in 21 cases.

The histology diagnostics were revised; the paraffin blocks of specimens were submitted to new cross-sections and to immunohistochemistry dyes for antibodies against fibronectin, laminin and collagen IV. Normal larynxes of five adult cadavers (three females and two males), without macroscopic lesions were used as controls in the immunohistochemistry study, after making sure these patients were non-smokers and had not been submitted to tracheal intubation.

### Immunohistochemical study

The paraffin blocks of the 60 patients were submitted to 3 micron cross-sections. The specimens were then placed on silanized slides and submitted to immunohistochemical reactions by the Avidin Biotin Peroxidase11 method, using anti-fibronectin polyclonal antibody (code A0245, Dako), monoclonal antibody (clone LAM 89, Novocastra) and anti-collagen IV monoclonal antibody (clone CIV 22, Dako). After preparing the material, the histology slides were examined under light microscopy (Zeis - Carl Zeiss do Brasil Ltda; Axiostar plus model), by an experienced pathologist, with different magnifications and image capture by a digital camera. Results were interpreted depending on the degree of color intensity into mild, moderate or intense. The immunoexpression sites were: basal membrane, corium and vessel endothelium. The normal larynxes used as control were submitted to the same immunohistochemical reactions.

### Statistical Method

For histology analysis, the statistical method used was the chi-squared, with a significance level of 5%.

## RESULTS

**Figure 1:**
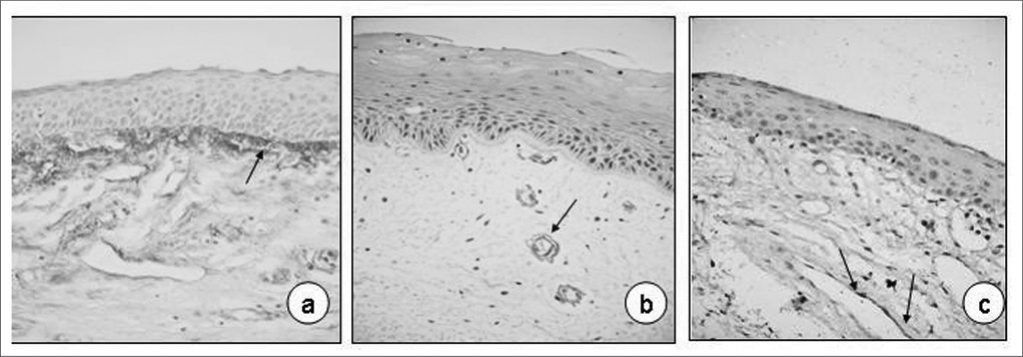
Normal vocal fold. Fibronectin immunoexpression on the basal membrane and lamina propria superficial layer (a 40X; arrows), collagen IV and laminin on the basal membrane of vessel epithelium (b and c, 40X; arrows).

Immunohistochemical analysis of the larynxes used as control was very similar in the five fragments of vocal folds assessed, in other words, we identified mild fibronectin pigmentation, especially on the basal membrane (Fig. 1a), although in the vessel endothelium and corium we also noticed the pigment, however less markedly. Collagen IV and laminin immunoexpression were clearer around the vessels (Figs. 1b and 1c).

**Figure 2:**
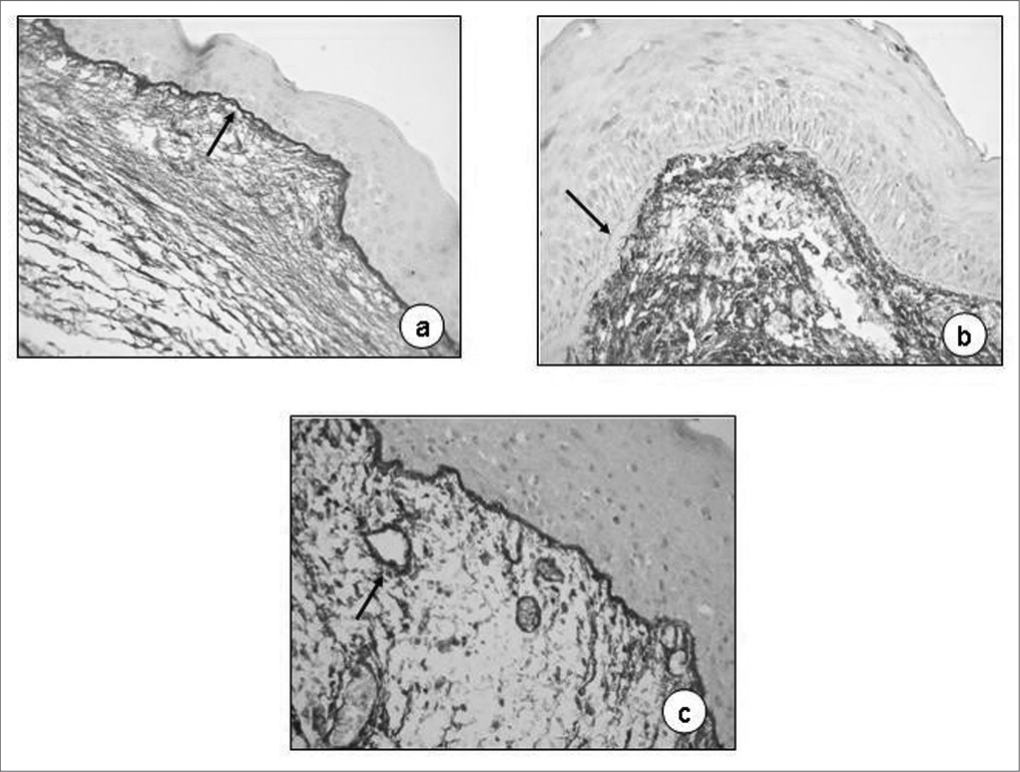
Reinke's edema. Fibronectin immunoexpression on the basal membrane (a, 40X; arrow) and on the basal membrane of the vessel endothelium (C, 60X; arrow). No pigment on the basal membrane on b (60X, arrow).

Table 1 shows the semi-quantitative scores of the immunohistochemical analyses of fibronectin on the 60 slides analyzed, where we can notice that the pigment deposit on the basal membrane and corium was not impressive (25.0% and 23.33%, respectively), similarly to the specimens used for control purposes, stressing the delicate brownish contour on the entire basal membrane (Fig. 2a). In some cases the basal membrane was completely free from pigment impregnation, even when thickened (Fig. 2b). In the lamina propria vessels' endothelium, there was marked impregnation, present in 68.33% of the cases (Fig. 2c).

**Table 1:** Fibronectin immunoexpression semiquantitative score in Reinke's edema.

	Fibronectin immunoexpression score					
Sites	0 absent	1 mild	2 moderate	3 Intense	Total N %	p
Basal membrane	45	13	2	0	15 (25,00)	<0,0001
Corium	46	13	1	0	14 (23,33)	<0,0001
Vessel endothelium	19	34	7	0	41 (68,33)	0,0001

**Figure 3:**
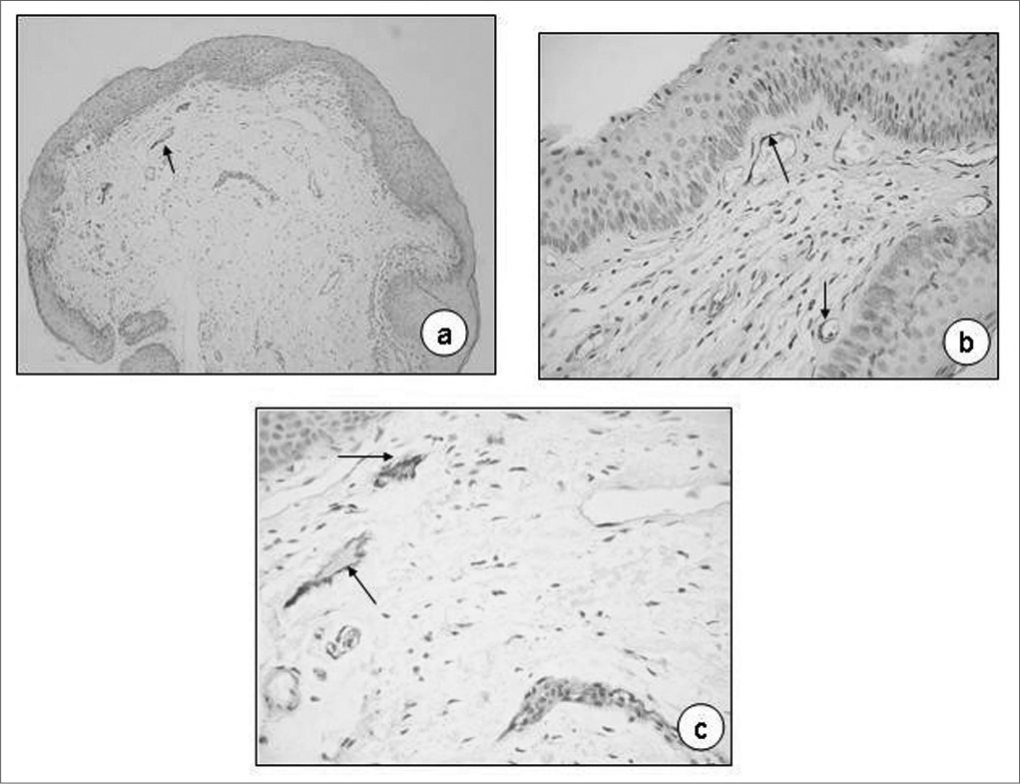
Reinke's edema. Laminin immunoexpression on the basal membrane of vessel endothelium (a, 20X; b, 40X; C, 80X; arrows).

Table 2 shows the results of the laminin immunoexpression analyses in Reinke's Edema on the 60 slides analyzed and, once again, we notice a greater deposit of pigment on the vessels' endothelium (73.33%; Figs. 3a, 3b and 3c); Anti-laminin antibodies were practically absent on the basal membrane; and we notice them in only 5% of the slides analyzed.

**Table 2:** Laminin immunoexpression semiquantitative score in Reinke's edema.

	Laminin immunoexpression score					
Sites	0 absent	1 mild	2 moderate	3 Intense	Total N %	p
Basal membrane	57	3	0	0	3 (5,00)	<0,0001
Corium	60	0	0	0	0 (0,00)	<0,0001
Vessel endothelium	16	39	5	0	44 (73,33)	<0,0001

**Figure 4:**
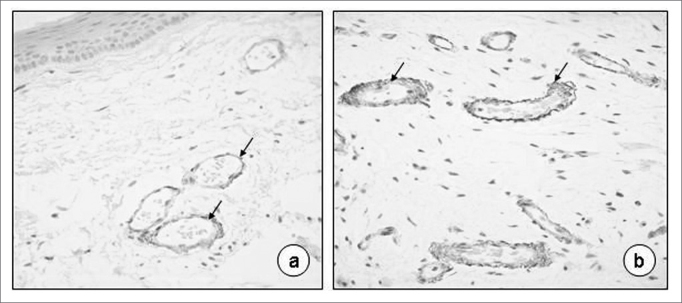
Reinke's edema. Collagen IV immunoexpression on the basal membrane of vessel endothelium (a-40X; b-80X; arrows).

On Table 3, we see the results of the collagen IV immunohistochemical analyses, and a predominance of the brownish color around the vessels (76.66%), present only in 3.33% on the basal membrane of the slides investigated (Figs. 4a and 4b).

**Table 3:** Collagen IV immunoexpression semiquantitative score in Reinke's edema.

	Collagen IV immunoexpression score					
Sites	0 absent	1 mild	2 moderate	3 Intense	Total N %	p
Basal membrane	58	2	0	0	2 (3,33)	<0,0001
Corium	59	1	0	0	1 (1,66)	<0,0001
Vessel endothelium	16	28	14	2	46 (76,66)	<0,0001

## DISCUSSION

Reinke's edema is considered benign for most of the authors, classified among exudative lesions on Reinke's space, together with vocal nodules and polyps^4^,^5^. Pastuszek et al.^12^, analyzed 261 slides with Reinke's edema previously dyed by hematoxylin-eosin, and stressed the high frequency of subepithelial edema and dilated vessels on the lamina propria, and less epithelial dysplasia. Concerning ultrastructural analyses, the authors stress the loss of intercellular junctions. Among other histological findings, we have inflammatory infiltrate and basal membrane thickness.

Because of the direct relationship between Reinke's edema and chronic smoking, it is important to carefully assess its morphological aspects. In a recently published study, we presented the histological findings and those from electron scanning and transmission microscopies of Reinke's edema, reporting on different levels of epithelial dysplasia and one case of in situ carcinoma^13^. This concern about possible pre-neoplastic alterations associated with Reinke's edema had already been reported by some authors^14^, ^15^. Marcotullio et al.^15^ studied 125 cases of Reinke's edema and found 52 cases classified as grade 0 epithelial alterations, 64 grade I, 6 grade II and 3 grade III, among which there was one case of microinvasive carcinoma.

Through immunohistochemical dyes using anti-fibronectin, anti-laminin and anti-collagen IV antibodies, the lamina propria vessels can be well highlighted, since they are in large number. In the present investigation we noticed that the anti-fibronectin antibodies deposit on the basal membrane was not a frequent finding in Reinke's edema. According to Gray et al.^6^ and Volic et al.^8^, the fibronectin deposit on the basal membrane is a marked characteristic of vocal polyps. Fibronectin is a glycoprotein produced by fibroblasts, having an important role to play on tissue repair and adhesion, which justifies its increase in speech-trauma lesions. For Reinke's edema, the speech overload does not seem to be the determining factor responsible for the development of the lesion, but rather the chronic irritative action from cigarette components, responsible for the inflammatory infiltrate and increase in the number of vessels. The resulting exudate is produced and stored in the lamina propria, since local lymphatic drainage is precarious.

Neves et al.^16^ carried out a histopathological and immunohistochemical study using antibodies against laminin and collagen IV in laryngeal lesions (9 nodules, 8 polyps and 9 Reinke's edema) and reported a greater immunoexpression of laminin and collagen IV on vocal nodules, as well as a greater thickening of the basal membrane when compared to polyps. Reinke's edema was not different from the other lesions studied. The authors stressed the important role played by these substances on speech trauma lesions; however, they did not study fibronectin, which response seems to be greater than that of laminin and collagen IV.

Additional studies have shown that besides the morphological alterations shown above, the distribution of collagen fibers seem to be also altered in Reinke's edema. Sakae et al.^17^ analyzed 20 cases of Reinke's edema using the picrosirius method and observed that the collagen fibers of the lamina propria lost their characteristic intertwining pattern, entering in disarray and rupturing, amidst a myxomatous infiltrate, especially of the deeper layers of the lamina propria. These alterations can contribute to the vocal dysfunction found among these patients.

As we have seen, the many morphological studies about Reinke's edema add information which help unveil the lesion pathophysiology and explain the alterations in the voice quality of these patients. Considering the immunoexpression of the basal membrane fibronectin, laminin and collagen IV did not show revelevance in immunohistochemical studies, these antibodies prevail in the endothelium of the lamina propria vessels.
